# Improving efficiency of parallel inverters operation in island mode microgrids

**DOI:** 10.1038/s41598-023-47679-4

**Published:** 2023-11-25

**Authors:** Mohamed Zaki, Ahmed Shahin, Saad Eskender, Mohamed A. Elsayes

**Affiliations:** Electrical Engineering Department, Faculty of Engineering, Mansoura University, Mansoura, Egypt

**Keywords:** Electrical and electronic engineering, Energy grids and networks, Power distribution

## Abstract

DC/AC inverters play a vital role in microgrids, efficiently converting renewable energy into usable AC power. Parallel operation of inverters presented numerous challenges, including maximizing system efficiency, minimizing circulating current, and maximizing system accuracy. This proposal introduces an analytical optimization technique designed to enhance the efficiency of paralleled inverters in microgrid systems while minimizing circulating current. The system parameter estimation is performed with a rapid recursive least squares (RLS) estimator. An optimized proportional-integral-derivative (PID) controller achieves high accuracy and streamlining system construction. The performance of the proposed optimizer is compared to common optimization methods, such as particle swarm optimization (PSO), neural networks, interior search, and interior point optimizers, focusing on system efficiency and eliminating circulating currents. Simulation investigations validated the method's applicability and demonstrated the proposed optimizer's superiority in efficiency, stability, and limiting circulating currents. It also achieved zero execution time, significantly outperforming alternatives like the neural network optimizer, which took 0.693 s. In various scenarios, the proposed optimizer improved system efficiency by 3% compared to the equally shared current system.

## Introduction

The increasing investment in renewable energy sources has created greater urgency for inverters to improve in terms of efficiency and dependability. Multiple inverters must be operated in parallel at peak efficiency to satisfy the frequency, voltage, and power quality requirements of loads with diverse characteristics and qualities^1,2^. Various academic articles have classified methods for controlling parallel inverters. These studies have divided control systems into two categories: centralized and decentralized^1,3–5^. The modules in parallel inverter systems are frequently dissimilar, which leads to an imbalance in the distribution of load current. Therefore, certain modules may be carrying an excessively large current.

Using parallel-operated inverters instead of a single centralized inverter improves system reliability, control, stability, and cost due to mass production^6,7^. Additionally, when integrating multiple renewable energy systems, it is essential to either link parallel inverters to the AC bus, as depicted in^8^, or to connect several DC-DC converters in parallel to the DC bus, as illustrated in^9^. The challenge is to exchange power across inverters while maximizing efficiency and limiting circulating currents. The study conducted by^10^ proposes a control method for parallel inverters that utilizes a modified sliding mode control coupled with the best Riccati control approach. This approach aims to improve the effectiveness of the parallel inverter system in microgrid systems. A redesigned droop-control technique using a virtual resistor scheme that regulates the power-sharing of inverters throughout a wide range is offered by^11^.

In the study conducted by^12^, a PSO-based droop controller is proposed to enhance the stability of paralleled inverters in microgrid systems. A droop control method based on fuzzy logic for parallel inverters is proposed in^13^. PSO is employed in^14^ to calculate the losses in parallel inverters by examining the efficiency curve fitting of a single inverter.^15^ creates mathematical models to evaluate the reliability of paralleled inverters, utilizing different topologies and control methods. One of the significant challenges of paralleling inverters is the occurrence of circulating currents between them. Numerous methods, such as^6,16–19^, have been proposed to eliminate circulating currents in parallel inverter systems. All these methods are designed to resolve a singular issue presented by parallel inverters. The main drawback of the mentioned methods is their complex control.

The most common method for regulating inverters is through PID control. PID control maintains desired output levels by adjusting inverter operating parameters based on error feedback. Incorporating fuzzy logic into a PID controller, often referred to as fuzzy-PID control, enables real-time dynamic adjustments to various environmental conditions^20–22^. Implementing fuzzy-PID control presents numerous challenges, including complexities in design, difficulties in the tuning process, increased computational load, and susceptibility to noise interference. Algorithmic enhancements, such as parameter tuning and predictive control algorithms, contribute to improving both PID control and inverter performance. This innovative control method guarantees stable and precise power conversion, rendering it a valuable tool in inverter technology for various applications^23,24^. Owing to its prior advantages, a PSO-based optimized PID controller will be designed to regulate the system’s inverters.

A master–slave controller will enhance system efficiency while minimizing circulating currents^25,26^. A simple analytical optimizer based on system parameters will also be proposed, along with a straightforward online estimator to estimate system losses. The master inverter regulates the output voltage via a PID voltage control loop. The controller continuously compares the measured output voltage with the reference signal to maintain the desired voltage level. The controller regulates power distribution among parallel inverters to ensure optimal efficiency. Synchronizing the currents of slave inverters with the master inverter eliminates circulating currents.

A review of related research reveals that prior studies have primarily concentrated on addressing specific issues, such as minimizing circulating currents, achieving equitable power distribution among inverters, maximizing system efficiency, or evaluating the reliability of paralleled inverters. These studies have typically relied on complex controller designs to address singular problems. This study aims to introduce an analytical optimization technique to maximize system efficiency while simultaneously minimizing circulating currents, all within a simplified control system.

The principal contribution of this research is the development of an optimizer designed to enhance the efficiency of parallel inverters and limit circulating currents. This analytical optimizer was developed based on Lagrange mathematical analysis of the system's loss function to achieve a streamlined and highly efficient optimization process compared to meta-heuristic optimization techniques used by^20,27,28^. The optimizer operates by leveraging the system's parameters to attain a state of maximum efficiency. The RLS estimator was chosen to estimate the system parameters promptly. This decision was based on its efficient computational capabilities and the straightforward parameter equation in D-Q Axis representation. These factors distinguish it from other estimators used in previous studies^21^. The proposed optimizer provides the following benefits: it ensures a 3% higher efficiency compared to equally shared current scenarios, significantly reduces execution time compared to alternatives (e.g., the neural network optimizer takes 693 ms), and facilitates a streamlined and rapid system control.

This paper proposes an analytical optimizer to increase the parallel inverter system’s efficiency. The primary objective is to establish a control scheme that maximizes the efficiency of paralleled inverters while simultaneously limiting circulating currents. The proposed approach involves a master–slave parallel inverter system that optimizes electrical power sharing between inverters to maximize system efficiency. A Lagrange analysis of the power losses equation for the system is conducted in the D-Q axes to derive the current values in which each inverter participates to have maximum efficiency. The optimizer utilizes an RLS estimator to estimate the system parameters required in the optimization equation rapidly. An optimized PID controller is employed to regulate the parallel inverter system. The results of PSO, Neural network, Interior search, and interior point optimizers are compared to those of the proposed optimizer. A simulation model of three parallel inverters is employed to evaluate the effectiveness of the proposed control system compared to other optimization techniques and to assess its performance relative to the unoptimized system. Three case studies are conducted using inverters with varying parameters and different loads.

## Paralleled ınverters model

Equation (1) utilizes the Park transformation to convert the voltages and currents of the three-phase inverter to stationary d-q axes. This simplifies the control system, allowing for separate control of active and reactive power and separate control of voltage and frequency^29^. After completing the control procedures, Eq. (2) converts the d-q values back to three-phase values. These values are then utilized to generate control signals for the inverters' power electronic switches.

1 $$\left[\begin{array}{c}{i}_{d}\\ {i}_{q}\\ {i}_{0}\end{array}\right]=\left[\begin{array}{ccc}sin(\mathrm{nwt})& cos(nwt)& 0\\ cos(nwt)& sin(nwt)& 0\\ 0& 0& 1\end{array}\right]\times \sqrt{\frac{2}{3}} \left[\begin{array}{ccc}1& -0.5& -0.5\\ 0& \frac{\sqrt{3}}{2}& -\frac{\sqrt{3}}{2}\\ \frac{1}{\sqrt{2}}& \frac{1}{\sqrt{2}}& \frac{1}{\sqrt{2}}\end{array}\right]\times \left[\begin{array}{c}{i}_{a}\\ {i}_{b}\\ {i}_{c}\end{array}\right]$$

2 $$\left[\begin{array}{c}{i}_{a}\\ {i}_{b}\\ {i}_{c}\end{array}\right]=\sqrt{\frac{2}{3}} \left[\begin{array}{ccc}sin(\mathrm{nwt})& cos(nwt)& \frac{1}{\sqrt{2}}\\ sin(\mathrm{nwt}+240\mathrm{n})& cos(nwt+240n)& \frac{1}{\sqrt{2}}\\ sin(\mathrm{nwt}+120\mathrm{n})& cos(nwt+120n)& \frac{1}{\sqrt{2}}\end{array}\right]\times \left[\begin{array}{c}{i}_{d}\\ {i}_{q}\\ {i}_{0}\end{array}\right]$$

**Figure 1:**
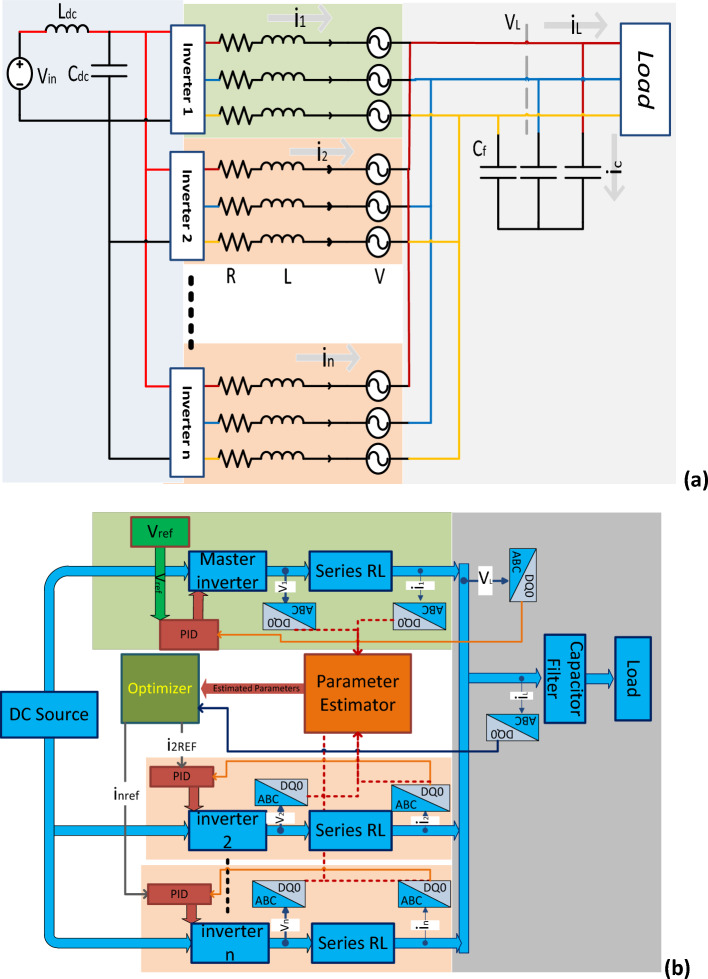
The system schematic: (**a**) the power system model, (**b**) the control system model.

According to Eqs. (3) and (4), the sum of the three-phase load currents and the three-phase currents of all N inverters in Fig. 1 is zero. At the start of operation, the capacitor output voltages and capacitive currents are both equal to zero. To simplify the model of parallel voltage-source inverters, Eq. (5) shows a stationary d-q-o form of the system. The voltage on the capacitive AC bus remains constant, meaning that the voltage component v_c0_ is always zero.

3 $${\mathrm{i}}_{\rm{La}}+{\mathrm{i}}_{\rm{Lb}}+{\mathrm{i}}_{\rm{Lc}}=0$$

4 $$\sum_{k=1}^{n}\left({i}_{ak}+{i}_{bk}+{i}_{ck}=0\right)$$

5 $$\frac{d}{dt}\left(\begin{array}{c}{V}_{cd}\\ {V}_{cq}\end{array}\right)=\left(\begin{array}{cc}0& \omega \\ -\omega & 0\end{array}\right)\left(\begin{array}{c}{V}_{cd}\\ {V}_{cq}\end{array}\right)+\frac{1}{{C}_{f}}\left(\left(\begin{array}{c}\sum_{k=1}^{n}{i}_{dk}\\ \sum_{k=1}^{n}{i}_{qk}\end{array}\right)-\left(\begin{array}{c}{i}_{Ld}\\ {i}_{Lq}\end{array}\right)\right)$$

Based on Eq. (3), the total system may have a zero-current component as it can flow through the paralleled diodes of the switches. The summation of zero component $\sum_{k=1}^{n}{i}_{0k}$ equals zero. Canceling the zero-sequence currents of the first inverter is required to cancel the zero-sequence currents of N-1 modules as ${i}_{01}=-\sum_{k=2}^{n}{i}_{0k}$^29^. The output currents d-q components of inverters’ current are shown in Eqs. (6) and (7).

6 $$\frac{d}{dt}\left(\begin{array}{c}{i}_{d1}\\ {i}_{q1}\end{array}\right)=\left(\begin{array}{cc}\frac{-{r}_{1}}{{L}_{1}}& \omega \\ -\omega & \frac{-{r}_{1}}{{L}_{1}}\end{array}\right)\left(\begin{array}{c}{i}_{d1}\\ {i}_{q1}\end{array}\right)+\frac{1}{{L}_{1}}\left(\left(\begin{array}{c}{V}_{d1}\\ {V}_{q1}\end{array}\right)-\left(\begin{array}{c}{V}_{td1}\\ {V}_{tq1}\end{array}\right)-\left(\begin{array}{c}{V}_{cd}\\ {V}_{cq}\end{array}\right)\right)$$

The remaining modules' inductive current K_th_, with k ∈ {2, …, n}, are:

7 $$\frac{d}{dt}\left(\begin{array}{c}{i}_{0k}\\ {i}_{dk}\\ {i}_{qk}\end{array}\right)=\left(\begin{array}{ccc}\frac{-{r}_{k}}{{L}_{k}}& 0& 0\\ 0& \frac{-{r}_{k}}{{L}_{k}}& \omega \\ 0& -\omega & \frac{-{r}_{k}}{{L}_{k}}\end{array}\right)\left(\begin{array}{c}{i}_{0k}\\ {i}_{dk}\\ {i}_{qk}\end{array}\right)+\frac{1}{{L}_{k}}\left(\left(\begin{array}{c}{V}_{0k}\\ {V}_{dk}\\ {V}_{qk}\end{array}\right)-\left(\begin{array}{c}{V}_{t0k}\\ {V}_{tdk}\\ {V}_{tqk}\end{array}\right)-\left(\begin{array}{c}{V}_{c0}\\ {V}_{cd}\\ {V}_{cq}\end{array}\right)\right)$$

where: the voltages v_tdk_, v_tqk_ with k 1, …, n are brought into the model as voltages drop and losses in inverter switches.

## Inverter’s optimized PID controller

The role of the controller is to ensure that the system operates at its optimal performance level. The proposed system is based on current sharing, with one inverter acting as the master to maintain a constant load bus voltage. All inverters collaborate in current sharing to minimize system losses, as demonstrated in Fig. 1. The system optimizer determines each inverter's contribution to the current value. A PID controller is employed to regulate each inverter, chosen for its quick response, ease of use, and extensive application in industrial processes. Additionally, the inverter switches are meticulously controlled through SPWM. The error signal, which represents the difference between the system's present state and its desired reference state, serves as the input for the PID control algorithm. The error signal is subjected to extraction, differentiation, and integration, with each operation being multiplied by a unique constant. The resulting values are combined using Eq. (8), which provides the input to the inverters.

8 $${u}_{t}={K}_{p}*{e}_{t}+{K}_{i}*\int {e}_{t}dt+{K}_{d}*\frac{d{e}_{t}}{dt}$$

where: ${K}_{p}$ is proportional constant, ${K}_{i}$ is the integration constant, and ${K}_{d}$ is the differentiation constant.

Equation (9) uses a filter for the differential component of the controller, which helps prevent system instability caused by noise. There are numerous techniques for determining the values of constants, such as Roth Horizon^30,31^.

PSO is utilized in the tuning of PID parameters, as described in^23,24,32,33^. PSO-based control substantially enhances system stability and precision by minimizing settling time and overshoot reduction. The PID constants for the inverter circuit are determined using its transfer function. Equation (10) shows the transfer function of the output voltage to the input voltage, while Eq. (11) shows the transfer function of the output current to the input voltage. The PID constants are determined using the PSO optimization technique. This is accomplished through two methods: the first method employs an offline approach utilizing the system transfer functions in Eqs. (10) and (11) to fine-tune the PID constants with PSO. The second method is an online approach that includes system simulation for tuning PID parameters using PSO. This paper will employ the first method.

9 $${u}_{s}={K}_{p}*{e}_{s}+{K}_{i}*\frac{1}{S}*{e}_{s}+{K}_{d}*\frac{S}{{T}_{f}*S+1}*{e}_{s}$$

10 $$\frac{{V}_{(t)}}{{u}_{(t)}}=\frac{SLR}{{(L{L}_{1}CR)S}^{3}+{(L{L}_{1}+LCR{R}_{1})S}^{2}+\left(LR+R{L}_{1}+L{R}_{1}\right)S+R{R}_{1}}$$

11 $$\frac{{i}_{(t)}}{{u}_{(t)}}=\frac{LCR{S}^{2}+LS+R}{{(L{L}_{1}CR)S}^{3}+{(L{L}_{1}+LCR{R}_{1})S}^{2}+\left(LR+R{L}_{1}+L{R}_{1}\right)S+R{R}_{1}}$$

where: R is the load equivalent resistance, L is the load equivalent inductance, C Load parallel capacitor filter, ${L}_{1}$ inverter series inductor filter, ${r}_{1}$ inverter series losses resistance, ${T}_{f}$ is the filter time constant.

## Optimization techniques

Optimization is a process aimed at identifying the optimal solution from a set of possible solutions. This process encompasses two primary categories. The first category, known as deterministic optimization, relies on mathematical analysis of the objective function to identify the optimal solution. The second category, stochastic optimization, involves using heuristics that leverage random variables to determine the next steps in the optimization process^34^. In this part, several well-known optimization methods will be introduced, with which the proposed method will be compared.

The interior point method is a well-known deterministic optimization algorithm for optimization problems with nonlinear constraints. This method is a modification of several traditional optimization techniques, including Newton's optimization method^35–37^. Hence, it will be employed in this study as a representation of deterministic methods.

PSO, a metaheuristic optimization method, offers superior parallel processing, robustness, and probability of retrieving global optimal solutions, outperforming random methods. The technique simulates a flock of birds searching for food using randomly imposed locations on particles. The particle that performs best relative to others stops, and the remaining particles move toward the optimal position at varying rates until one of them reaches the best value. They then move towards the new optimal position, and this process is repeated until all particles converge on a single point representing the optimum value of the optimized function^38–40^. Particles begin at random locations and move to new locations at specific speeds based on Eq. (12). The speed of each particle is determined by its previous speed, its position relative to the best location it has reached, and its proximity to the best particle in the swarm, as described in Eq. (13).

12 $${{P}_{i}}^{t+1}={{P}_{i}}^{t}+{{v}_{i}}^{t+1}$$

13 $${{v}_{i}}^{t+1}=w*{{v}_{i}}^{t}+{c}_{1}*{r}_{1}\left({{P}_{local best\left(i\right)}}^{t}-{{P}_{i}}^{t}\right)+{c}_{2}*{r}_{2}\left({{P}_{global best\left(i\right)}}^{t}-{{P}_{i}}^{t}\right)$$

Where: ${P}_{i}$ position of $i$ particle, ${v}_{i}$ speed of $i$ particle, $w$ inertia weight, ${P}_{local best\left(i\right)}$ the best position of I particle, ${P}_{global best\left(i\right)}$ the best position in the swarm, ${r}_{1}{\& r}_{2}$ random numbers, ${c}_{1}\& {c}_{2}$ acceleration coefficients.

The interior-search method follows the same principle as the previous method of randomly distributing particles and comparing their values to arrive at the optimal solution. This approach involves randomly dispersing particles within the function's limits and dividing them into three regions. The particle that achieves the highest function value is deemed the global best, and the other particles are split into two groups. The Mirrored Group, which is close to the global best, is dispersed near it to find a better solution, while the remaining particles are randomly dispersed in the remaining space^41^. Equation (14) updates the third group's elements through a random distribution within the function's limits during each iteration. The mirror group is updated near the global best point randomly using Eq. (15). The global best point undergoes slight movements, as described in Eq. (16), while Eq. (17) ensures that no position is modified between iterations unless the element's value in the new iteration is superior to that of the prior iteration.

14 $${{x}_{i}}^{j}={LB}^{j}+({UB}^{j}-{LB}^{j})*{r}_{2}$$

15 $${{x}_{i}}^{j}=2*({r}_{3}*{{x}_{i}}^{j-1}+(1-{ r}_{3})*{{x}_{gb}}^{j})-{{x}_{i}}^{j-1}$$

16 $${{x}_{gb}}^{j}={{x}_{gb}}^{j-1}+{r}_{n}*\Upsilon$$

17 $${{x}_{i}}^{j}=\left\{\begin{array}{c}{{x}_{i}}^{j}\,if({{x}_{i}}^{j}>{{x}_{i}}^{j-1})\\ {{x}_{i}}^{j-1}\end{array}\right.$$

where: $\Upsilon$ is a value of around 0.01.

The artificial neural network simulates the function of neural networks in the human body. The commonly used feed-forward type is designed for a control process that employs fixed weighted values to attain a predetermined solution. The recurrent neural network optimization algorithm modifies weighted functions to find the optimal solution by initially assuming random input values and weights. The system output is fed back to the input until the optimal function value is achieved^42^. Equation (18) enables the neurons to select new x values and compute function values using the random values for particles and weight functions in Eq. (19). Weight functions move towards the optimal weight through Eq. (20).

18 $${{x}_{i}}^{t+1}={{x}_{i}}^{t}+{{x}_{i}}^{new(t+1)}$$

19 $${{x}_{1}}^{new(t+1)}={w}_{11}*{{x}_{1}}^{t}+{w}_{21}*{{x}_{2}}^{t}+{w}_{31}*{{x}_{3}}^{t}+... +{w}_{n1}*{{x}_{n}}^{t}$$

20 $${{w}_{i}}^{t+1}={{w}_{i}}^{t}+2*rand*({{w}_{best}}^{t}-{{w}_{i}}^{t})$$

## Proposed optimization technique

The analytical method for achieving the highest efficiency in a parallel-connected group of inverters relies on minimizing system losses through a function of system parameters. These parameters include the equivalent resistance of the line losses between the inverters and the load, as well as the inductances of the low-pass filter and the on-state voltage drop of the inverter switches. This analytical optimization method is developed based on the d-q loss analysis of the system. The derivation begins by considering two parallel DC sources with series resistors and then proceeds to minimize losses using the constrained Lagrange equation. The second step involves applying the same method using two parallel AC sources with series of resistive and inductive losses. In the third step, a series AC voltage component is introduced to reduce Lagrange losses. Finally, the equation is generalized to accommodate any number of parallel inverter systems.

**Figure 2:**
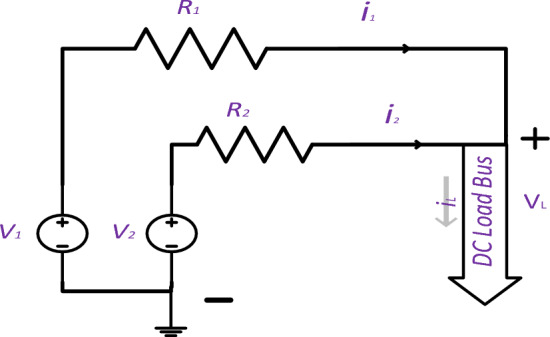
Two parallel DC sources feed a DC load.

First step: Fig. 2 shows a DC circuit with two discrete sources (V_1_ and V_2_) supplying the DC bus through two lines with resistances (R_1_ and R_2_). To achieve constant load voltage (V_L_) and deliver Power P with minimal losses, Eq. (21) can be optimized using Lagrange Eq. (23) while ensuring that the sum of currents i_1_ and i_2_ equals the load current i_L_. Equation (24) shows the optimal current values, indicating that the system performs best when the load current is divided between two sources, similar to a current divider. Furthermore, losses are minimized when the two sources have equal voltage. Parallel systems of interconnected sources operate optimally when their voltages are identical, and their shared current behaves as if they were connected to a single source.

21 $${P}_{loss}={{i}_{1}}^{2}*{R}_{1}+{{i}_{2}}^{2}*{R}_{2}$$

22 $${i}_{1}+{i}_{2}={i}_{L}$$

23 $$L={{i}_{1}}^{2}*{R}_{1}+{{i}_{2}}^{2}*{R}_{2}+\lambda ({{i}_{L}-i}_{1}-{i}_{2} )$$

24 $$\left\{\begin{array}{c}{i}_{1}=\frac{{R}_{2}}{{R}_{2}+{R}_{1}}*{i}_{L} \\ {i}_{2}=\frac{{R}_{1}}{{R}_{2}+{R}_{1}}*{i}_{L}\end{array}\right.$$

**Figure 3:**
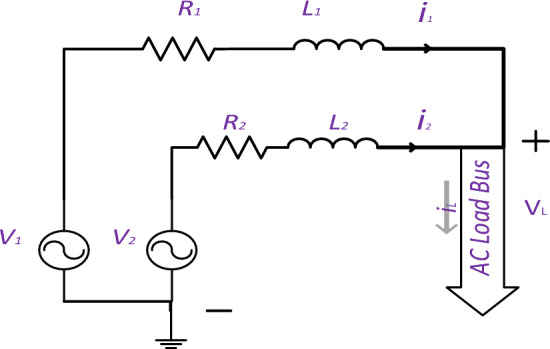
Two parallel AC sources feds AC load.

Second step: In Fig. 3, the parallel inverter circuit can be simplified by utilizing two AC sources that feed the AC bus via two lines, each with its own inductance and resistance values. By paralleling the capacitance with the load, the current value of the capacitance can be added to the load current. The line resistance and overall losses determine the value of resistance in the inverter, whereas the AC filter determines the values of inductance and capacitance. To minimize power losses in a parallel system, it is essential to identify the lowest value of power losses in Eq. (25) while adhering to the requirement in Eq. (26). The Lagrange Eq. (27) can be utilized to solve the optimization equation.

25 $${P}_{loss}={{i}_{1}}^{2}*{R}_{1}+{{i}_{2}}^{2}*{R}_{2}$$

26 $${i}_{1}+{i}_{2}={i}_{L}$$

27 $$L={{i}_{1}}^{2}*{R}_{1}+{{i}_{2}}^{2}*{R}_{2}+\lambda ({{i}_{L}-i}_{1}-{i}_{2} )$$

Equation (28) expresses the alternating current using magnitude and phase angle. Equation (29) splits the Polar Form into two values, one on the real axis and one on the imaginary axis. Equation (30) can be used to rewrite the Lagrange equation and obtain the desired result. By utilizing Eq. (30), it was discovered that the minimum value (provided in Eqs. (31), (32) and (33)) is proportional to the current divider values in the DC case. The results are unaffected by the current type or the presence of inductance. As the phase shift of the currents equals the load current, there is no circulating current between the parallel sources. In AC, the voltage is determined by multiplying the current with the total impedance of the line, and each source necessitates a distinct voltage level.

28 $$i*\mathit{sin}\left(wt-\varnothing \right)=i\mathrm{\angle \varnothing }$$

29 $$i {\angle \varnothing }={i}_{d}+{j*i}_{q}$$

30 $$L={{i}_{1d}}^{2}*{R}_{1}+{{i}_{2d}}^{2}*{R}_{2}+{{i}_{1q}}^{2}*{R}_{1}+{{i}_{2q}}^{2}*{R}_{2}+{\lambda }_{1}\left({{i}_{Ld}-i}_{1d}-{i}_{2d} \right)+{\lambda }_{2}\left({{i}_{Lq}-i}_{1q}-{i}_{2q} \right)$$

31 $$\left\{\begin{array}{c}{i}_{1d}=\frac{{R}_{2}}{{R}_{2}+{R}_{1}}*{i}_{Ld}\\ {i}_{1q}=\frac{{R}_{2}}{{R}_{2}+{R}_{1}}*{i}_{Lq}\end{array}\right.$$

32 $$\left\{\begin{array}{c}{i}_{2d}=\frac{{R}_{1}}{{R}_{2}+{R}_{1}}*{i}_{Ld} \\ {i}_{2q}=\frac{{R}_{1}}{{R}_{2}+{R}_{1}}*{i}_{Lq}\end{array}\right.$$

33 $$\left\{\begin{array}{c}{i}_{1}\angle {\varnothing }_{1}=\frac{{R}_{2}}{{R}_{2}+{R}_{1}}*{i}_{L}\angle {\varnothing }_{L} \\ {i}_{2}\angle {\varnothing }_{2}=\frac{{R}_{1}}{{R}_{2}+{R}_{1}}*{i}_{L}\angle {\varnothing }_{L}\end{array}\right.$$

Assuming that voltage is the result of dividing current by conductivity, Eq. (33) can be transformed into a function of conductivity, which is demonstrated in Eq. (35).

34 $$\left\{\begin{array}{c}{Y}_{1}=\frac{1}{{R}_{1}}\\ {Y}_{2}=\frac{1}{{R}_{2}}\\ \vdots \\ {Y}_{n}=\frac{1}{{R}_{n}}\end{array}\right.$$

35 $$\left\{\begin{array}{c}{i}_{1}\angle {\mathrm{\varnothing }}_{1}=\frac{ {Y}_{1} }{{Y}_{t}}*{i}_{L}\angle {\mathrm{\varnothing }}_{L} \\ {i}_{2}\angle {\mathrm{\varnothing }}_{2}=\frac{ {Y}_{2} }{{Y}_{t}}*{i}_{L}\angle {\mathrm{\varnothing }}_{L}\\ \vdots \\ {i}_{n}\angle {\mathrm{\varnothing }}_{n}=\frac{ {Y}_{n} }{{Y}_{t}}*{i}_{L}\angle {\mathrm{\varnothing }}_{L}\end{array}\right.$$

36 $${Y}_{t}=\frac{1}{{R}_{t}}={Y}_{1}+{Y}_{2}+\dots +{Y}_{n}$$

When considering a three-phase system, the ABC system can be transformed into the d-q-0 axis, representing the DC equivalent of a three-phase system. The Lagrange equations, when resolved for a three-phase system, yield the same results as those obtained for the D and Q currents in Eq. (32). If the number of parallel inverters is increased, it can be inferred that the optimal current in each parallel branch is equivalent to the current distribution in the parallel DC circuit. This is demonstrated in Eqs. (38) and (39).

**Figure 4:**
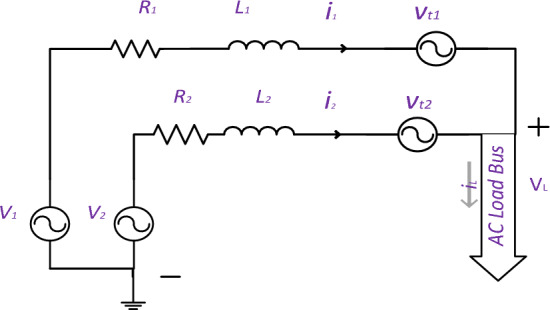
Two parallel AC sources feds a load through a series of resistive inductive and source losses.

Third step: a collection of parallel inverters does not limit the components of each inverter circuit to resistors and inductors. Figure 4 illustrates that the conduction losses of power electronic switches can be computed by multiplying the current passing through the switch with its on-state voltage drop. Hence, Eq. (37) is changed by modifying the Lagrange equations to include a new component. Solving Eq. (37) determines the current based on the resistor ratio. It introduces a novel current fraction added to one branch and subtracted from the other, depending on the difference in on-state voltage drop between the two sources. This is demonstrated in Eqs. (38) and (39).

37 $$L={{i}_{1d}}^{2}*{R}_{1}+{{i}_{2d}}^{2}*{R}_{2}+{{i}_{1q}}^{2}*{R}_{1}+{{i}_{2q}}^{2}*{R}_{2}+{v}_{t1d}*{i}_{1d}+{v}_{t2d}*{i}_{2d}+{v}_{t1q}*{i}_{1q}+{v}_{t2q}*{i}_{2q}+{\lambda }_{1}\left({{i}_{Ld}-i}_{1d}-{i}_{2d} \right) +{\lambda }_{2}\left({{i}_{Lq}-i}_{1q}-{i}_{2q} \right)$$

38 $$\left\{\begin{array}{c}{i}_{1d}=\frac{ {Y}_{1} }{{Y}_{t}}*{i}_{Ld} + \frac{{v}_{t2d}-{v}_{t1d}}{2*\left({R}_{2}+{R}_{1}\right)} \\ {i}_{1q}=\frac{ {Y}_{1} }{{Y}_{t}}*{i}_{Lq}+\frac{{v}_{t2q}-{ v}_{t1q}}{2*\left({R}_{2}+{R}_{1}\right)}\end{array}\right.$$

39 $$\left\{\begin{array}{c}{i}_{2d}=\frac{ {Y}_{2} }{{Y}_{t}}*{i}_{Ld} +\frac{{v}_{t1d}-{v}_{t2d}}{2*\left({R}_{2}+{R}_{1}\right)} \\ {i}_{2q}=\frac{ {Y}_{2} }{{Y}_{t}}*{i}_{Lq}-\frac{{v}_{t1q}-{ v}_{t2q}}{2*\left({R}_{2}+{R}_{1}\right)}\end{array}\right.$$

Final step: Eqs. (40) and (41) represent a generalization for systems containing more than two parallel inverters. The analysis reveals that the amount of current shared among the inverters to minimize system losses depends on both the system's resistances and the state voltage drop of the switches. Drawing upon the analysis conducted previously, the optimizer is tasked with estimating various inverter parameters, such as resistance and on-state voltage drop, to ascertain the optimal shared current value of each inverter.

40 $$\left\{\begin{array}{c}{i}_{1d}=\frac{ {Y}_{1} }{{Y}_{t}}*{i}_{Ld}+ \frac{{v}_{t2d}-{v}_{t1d}}{2*\frac{{y}_{t}}{{y}_{1}{*y}_{2}}}+\dots +\frac{{v}_{tnd}-{v}_{t1d}}{2*\frac{{y}_{t}}{{y}_{1}{*y}_{n}}}\\ {i}_{1q}=\frac{ {Y}_{1} }{{Y}_{t}}*{i}_{Lq}+\frac{{v}_{t2q}-{ v}_{t1q}}{2*\frac{{y}_{t}}{{y}_{1}{*y}_{2}}}+\dots +\frac{{v}_{tnq}-{v}_{t1q}}{2*\frac{{y}_{t}}{{y}_{1}{*y}_{n}}}\end{array}\right.$$

41 $$\left\{\begin{array}{c}{i}_{2d}=\frac{ {Y}_{2} }{{Y}_{t}}*{i}_{Ld}+ \frac{{v}_{t1d}-{v}_{t2d}}{2*\frac{{y}_{t}}{{y}_{1}{*y}_{2}}}+\dots +\frac{{v}_{tnd}-{v}_{t2d}}{2*\frac{{y}_{t}}{{y}_{2}{*y}_{n}}}\\ {i}_{2q}=\frac{ {Y}_{2} }{{Y}_{t}}*{i}_{Lq}+\frac{{v}_{t1q}-{ v}_{t2q}}{2*\frac{{y}_{t}}{{y}_{1}{*y}_{2}}}+\dots +\frac{{v}_{tnq}-{v}_{t2q}}{2*\frac{{y}_{t}}{{y}_{2}{*y}_{n}}}\end{array}\right.$$

## Parameter estimation techniques

The system state estimator determines the system's state based on inputs and outputs. The estimated state value serves as the actual feedback to the controller.^43,44^. The least-square method is one of the most simple and rapid approaches for estimating parameter values^44–46^. This method determines the inverter parameters by measuring its voltage and current. To estimate these parameters, the least-squares method, Eq. (42), is applied using the inverter model given in Eq. (7)^45^.

42 $$\left[\beta \right]={({{\psi }_{t}}^{T}*{\psi }_{t})}^{-1}*{{\psi }_{t}}^{T}\left[{Y}_{measured}\right]$$

The $\beta$ parameter matrix is obtained by differentiating the output equation by the system parameters for several readings, logging them in the ${\psi }_{t}$ matrix, and then forming the output measured values matrix ${Y}_{measured}$. Then, apply Eq. (42) to the system. The output voltage matrix is determined by calculating the voltage difference between the inverter and the load. It is a common practice to mitigate the on-state voltage drop of switches by treating them as V_t_ and consolidating all inductances and resistors within the inverter circuit into single components denoted as L and R, respectively.

43 $$\left\{\begin{array}{c}\left[\beta \right]=\left[\begin{array}{c}\mathrm{R}\\ L\\ {V}_{t}\end{array}\right]\\ \left[{\psi }_{t}\right]=\left[\begin{array}{ccc}\frac{d({V}_{dk}-{V}_{cd})}{dR}& \frac{d({V}_{dk}-{V}_{cd})}{dL}& \frac{d({V}_{dk}-{V}_{cd})}{d{V}_{t}}\\ \vdots & \vdots & \vdots \\ \frac{d({V}_{dk}-{V}_{cd})}{dR}& \frac{d({V}_{dk}-{V}_{cd})}{dL}& \frac{d({V}_{dk}-{V}_{cd})}{d{V}_{t}}\end{array}\right]=\left[\begin{array}{ccc}{i}_{d1}& \frac{d{i}_{d1}}{dt}-w*{i}_{q1}& 1\\ \vdots & \vdots & \vdots \\ {i}_{dn}& \frac{d{i}_{dn}}{dt}-w*{i}_{qn}& 1\end{array}\right]\\ \left[{Y}_{measured}\right]=\left[\begin{array}{c}\left({V}_{dk1}-{V}_{cd1}\right)\\ \vdots \\ \left({V}_{dkn}-{V}_{cdn}\right)\end{array}\right]\end{array}\right.$$

The offline least-square method relies on multiple output measurements for determining parameter values. An online estimation method with restricted memory usage would be required for the proposed system to be effective. The RLS method is a modified version of the least-square method that utilizes online parameter estimation^47–49^. The preference for RLS over many other estimation methods is attributed to its rapid adaptation, efficient memory utilization, and low computational latency. The following equations are the conclusion of the recursive least-square method used in online estimation. A new matrix ${f}_{k}$ is formed as in Eq. (44) to determine the RLS. In Eq. (45), the matrix ${f}_{k}$ is decomposed to extract the recent data from all the matrix data. Equation (46) represents the least-squares equation as a function of the ${f}_{k}$ matrix. To retrieve current data from all the stored data, a decomposition is also made to the ${Y}_{measured}$ matrix and the ${\psi }_{k}$ the matrix as shown in Eq. (47). Equation (49) illustrates the RLS method.

44 $${f}_{k}={{\psi }_{k}}^{T}*{\psi }_{k}$$

45 $${f}_{k}=\left[{{\psi }_{k-1}}^{T} {{\psi }_{k}}^{T}\right]\left[\begin{array}{c}{\psi }_{k-1}\\ {\psi }_{k}\end{array}\right]={\psi }_{k-1}*{\psi }_{k-1}+{{\psi }_{k}}^{T}*{\psi }_{k}$$

46 $$\left[\beta \right]={{f}_{k}}^{-1}*{\psi }^{T}\left[{Y}_{measured}\right]$$

47 $$\left[\beta \right]={{f}_{k}}^{-1}*\left[{{\psi }_{k-1}}^{T} {{\psi }_{k}}^{T}\right]\left[\begin{array}{c}{Y}_{k-1}\\ {Y}_{k}\end{array}\right]={{f}_{k}}^{-1}*\left({{\psi }_{k-1}}^{T}*{Y}_{k-1}+{{\psi }_{k}}^{T}*{Y}_{k}\right)={{f}_{k}}^{-1}*({f}_{k-1}*{\beta }_{k-1}+{{\psi }_{k}}^{T}*{Y}_{k}$$

48 $$\left[\beta \right]={{f}_{k}}^{-1}*(({f}_{k}-{{\psi }_{k}}^{T}*{\psi }_{k})*{\beta }_{k-1}+{{\psi }_{k}}^{T}*{Y}_{k})$$

49 $$\left\{\begin{array}{c}\therefore \left[\beta \right]={\beta }_{k-1}+{{f}_{k}}^{-1}*{{\psi }_{k}}^{T}({Y}_{k}-{\psi }_{k}*{\beta }_{k-1})\\ \therefore {f}_{k}={f}_{k-1}+{{\psi }_{k}}^{T}*{\psi }_{k}\end{array}\right.$$

## System model

A simulation model was developed in MATLAB to assess the effectiveness of the proposed controller. Figure 1 depicts the subject model, which features three parallel-connected inverters powered by a DC source via an LC filter. The output, shown in Fig. 1a, is also connected via an LC filter. The system comprises one master inverter and multiple slave inverters, as shown in Fig. 1. The master inverter maintains the load voltage at a constant set point. In contrast, the slave inverters work together to supply the load with a portion of power according to the reference values set by the system's main controller.

The system controller's main parts are shown in Fig. 1b. Each inverter has its own current and voltage sensors, as well as its own PID controller. The current and voltage of the load, as well as the capacitor filter current, are measured. All the data measured is represented on a d-q form.

The system controller consists of two primary components: the system's main controller, which employs an optimizer, and the PID current loop controller of each inverter. The optimizer's role is to determine the optimal reference currents across the three inverters to minimize power losses within the system. Diverse optimizers, including PSO, Neural Network, Interior Search, and Interior Point, are utilized to assess the comparative efficacy of the proposed optimizer.

The RLS estimator, using the estimator function in Eq. (49), calculates the values of inverter parameters. The estimator employs the inverter d-q model presented in Eq. (7) to calculate these parameters. The optimizer utilizes the estimated parameters to set reference values to minimize power losses within the system. The optimizer determines the reference d-q inverter currents using Eq. (41), whereas the compared optimizers calculate the reference d-q currents using Eq. (50), considering the system losses.

50 $${F}_{opt}=\sum_{k=1}^{n}\left({V}_{tdn}{i}_{dn}+{V}_{tqn}{i}_{qn}+{r}_{f}\left({i}_{dn}^{2}+{i}_{qn}^{2}\right)\right)$$

**Figure 5:**
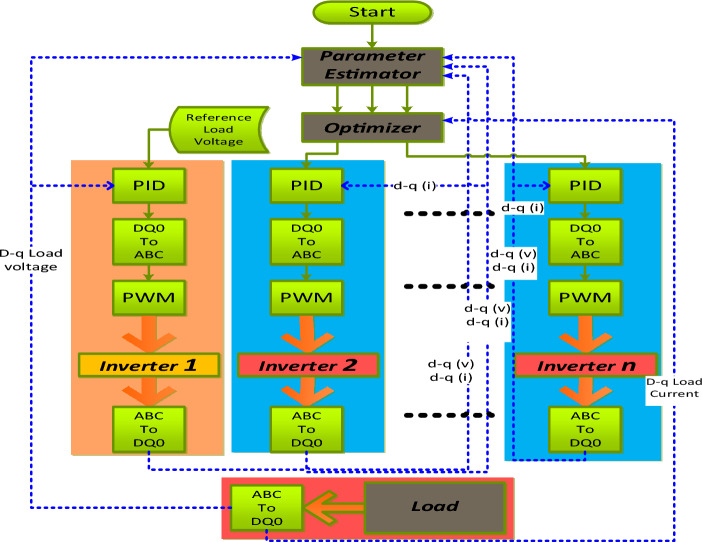
The proposed control system flowchart.

Figure 5 illustrates the system flowchart, which outlines the sequence of system processes. The data enters the system in the following order: first, the estimator estimates the system parameters based on the measured data. Next, the optimizer distributes the load current among the inverters according to the parameter values and sends the reference values to each inverter's controller. Each inverter controller confirms that the current passing through the inverter matches the reference value. For the master inverter, its current automatically equals the difference between the load current and the total currents of the slave inverters.

**Figure 6:**
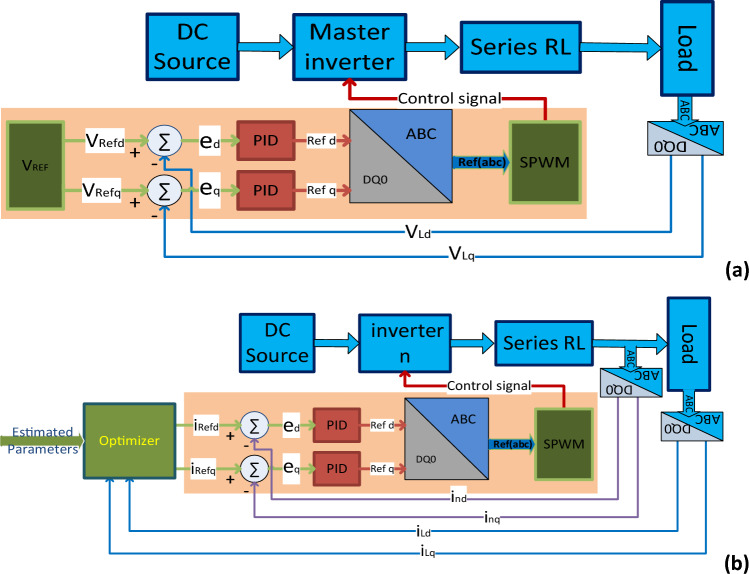
Inverter’s controller: (**a**) master inverter controller, (**b**) slave inverter controller.

Figure 6a illustrates the master inverter controller, which consists of a d-q PID voltage loop controller. The input signal to the controller is the voltage difference between the d-q reference voltages and the measured load voltage. The controller reference signals are converted to the ABC axes by utilizing a d-q to ABC transformer. The controller drives the inverter using SPWM. To optimize the controller's performance, PSO is employed to fine-tune the proportional, derivative, integrator, and differential filter coefficients that constitute the PID controller.

The current loop PID controller for the slave inverter, depicted in (b) of Fig. 6, compares the reference d–q current values generated by the optimizer with the actual d–q currents measured by the inverter. The output signals from the controller in d–q format are transformed into ABC axes. An SPWM then utilizes these signals to control the switches of the inverters.

## Simulation results

Three case studies were conducted to evaluate the system's effectiveness. These studies involved the use of inverters with varying parameters and load configurations. The primary objectives were to assess the proposed optimizer's ability to achieve maximum efficiency in comparison to other optimizers and to evaluate the control system's capacity to maintain system reliability and robustness.

Four optimizers are utilized and compared against the proposed methodology. The first optimizer is the interior search optimizer, with a population size of 40 and a maximum iteration count of 500. The second optimizer uses a recursive neural network algorithm with a population size of 50 and a maximum iteration count of 1000. The third optimizer is a PSO optimizer with 50 particles and a maximum iteration count of 500. The fourth optimizer is the built-in interior-point-constrained optimizer in MATLAB. The outputs of these four optimizers are then compared to the proposed analytical optimizer.

### Case 1

The first case involves three inverters with parameters listed in Table 1. The load varies from 3 kW between 0 and 80 ms to 5 kW between 80 and 200 ms, with a reactive power of 100 VAR. Table 2 displays the average power losses, revealing that the proposed method exhibits the lowest power losses. The columns in the table indicate the system's power losses. The first column shows the data for current equally distributed among the inverters without optimization. In contrast, the other columns exhibit the losses incurred when using PSO, Interior Search, Neural Network, Interior Point, and the proposed optimizer sequentially.

**Table 1:** Case 1 inverters’ parameters.

System resistance and reactance	Inverter 1	Inverter 2	Inverter 3	DC input filter
Series resistance	0.7 Ω	1.4 Ω	1 Ω	0.5 Ω
Inductance	1 mH	3 mH	2 mH	0.4 mH
Voltage drop	1.6 V	3.2 V	1.6 V	–-
Capacitance	–-	–-	–-	800 µF

**Table 2:** Case 1 system losses in the case of different optimizers.

Load power		Without optimization	PSO	Interior search	Neural network	Interior point	Proposed
3 kW	P_L_(w)	438	404.4	409.7	390	398	380
100 VAR	η (%)	87.2%	88.1%	87.9%	88.5%	88.3%	88.8%
5 kW	P_L_(w)	737	688	647	643.5	649	633.7
100 VAR	η (%)	87.2%	87.9%	88.5%	88.6%	88.5%	88.8%
7 kW	P_L_(w)	1119	1037	983.7	981	984	956.7
500 VAR	η (%)	86.2%	87.1%	87.7%	87.7%	87.7%	88%
10 kW	P_L_(w)	2000	3289	1780	1761	1758	1699
500 VAR	η (%)	83.3%	75.2%	84.9%	85%	85%	85.5%
Optimizer execution time			464.9 ms	342.6 ms	693.4 ms	7.7 ms	

When employing the proposed optimizer, power loss at a 3-kW load is minimized to 380 W, surpassing all other methods. Equal current sharing among inverters yields losses of 438 W, while other optimizers fall within this range. The table ranks the second-best optimizer as a neural network, with losses of 390 W, and the least efficient optimizer as the interior search method, resulting in losses of 409.7 W.

When a load of 7 kW is applied from 0 to 0.1 s, followed by a change to 10 kW until 0.2 s, the proposed method consistently produces the best results. In one case, the neural network optimizer comes in second, while in the other case, the interior point optimizer is the runner-up. However, when a 10 kW, 500 VAR load is connected, all other optimizers generate results close to the optimal value, except for the PSO method, which produces an incorrect value of 3289 W. The proposed optimizer yields the lowest power loss of 1699 W among all tested approaches at a 10-kW load. In contrast, using the equal current technique results in losses of 2000 W. The power losses of other optimizers fall between these two ranges. The table shows that the interior point is the second-best optimizer, with power losses of 1758 W.

**Figure 7:**
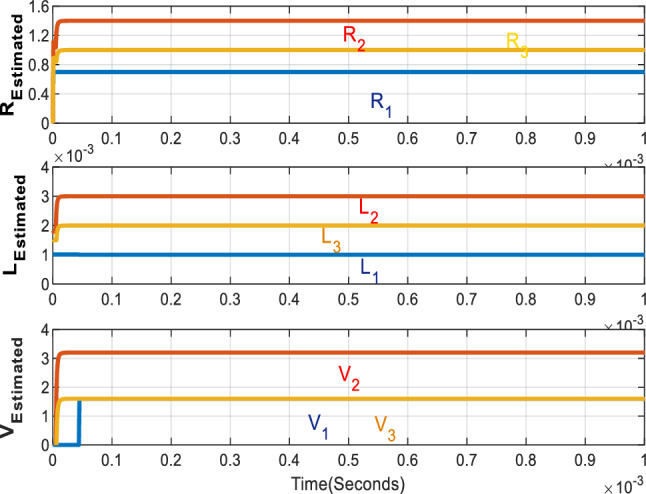
Inverters estimated parameters.

**Figure 8:**
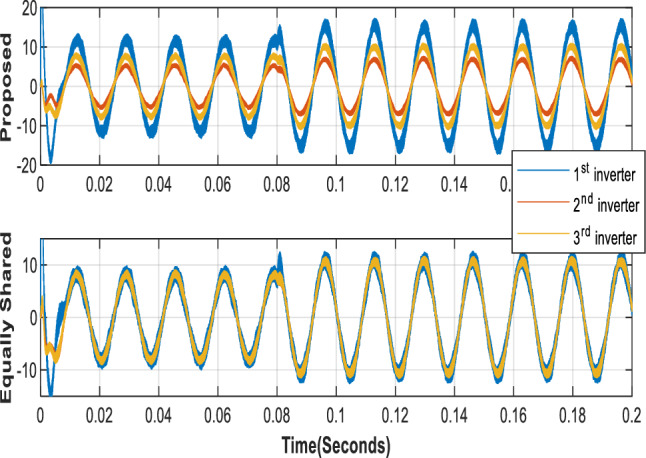
3 kW and 5 kW Phase (A) inverters currents.

In all cases, the fast RLS estimator accurately estimates the resistance, inductance, and voltage drop values in less than 100 microseconds, as illustrated in Fig. 7. Figure 8 compares the proposed optimizer with the equal current distribution approach based on phase (A) current. When the current is equally distributed, the three inverters carry identical currents. In contrast, the proposed method allocates current according to the optimizer's choices.

**Figure 9:**
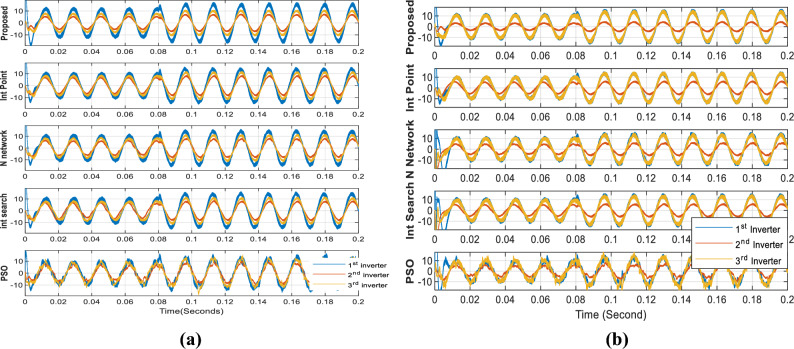
Phase (A) currents of the three inverters using the different optimizers at 3 kW and 5 kW loads (**a**) case 1 currents, (**b**) case 2 currents.

**Figure 10:**
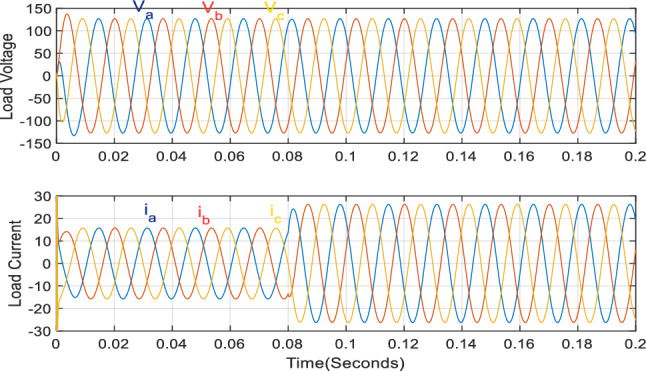
Load voltage and current in case of 3 kW and 5 kW.

Figure 9a compares the output phase A currents for all optimizers. In this scenario, all the inverters' phase A currents exhibit the same phase shift, which prevents circulating currents between them. The proposed optimizer generates the most stable currents, whereas the PSO optimizer produces unstable currents. The high-quality sine wave signals for both the load voltage and current, as shown in Fig. 10, are tuned by the LC filter.

**Figure 11:**
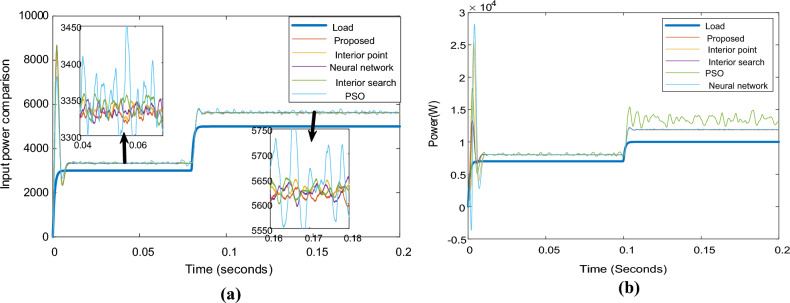
Systems input and output powers in case of different optimizers: (**a**) 3 kW and 5 kW load, (**b**) 7 kW and 10 kW load.

**Figure 12:**
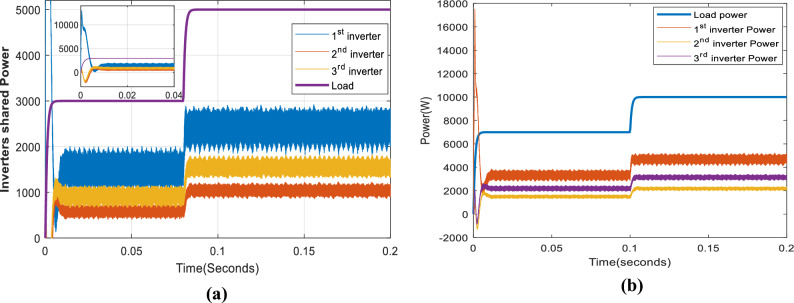
The three inverters shared powers: (**a**) in case of 3 kW and 5 kW load, (**b**) in the case of 7 kW and 10 kW.

Figure 11 depicts the input power of the system in the case of different optimizers. As demonstrated, although all optimizers have input powers within the same range, the proposed optimizer has the lowest input power relative to the others. PSO has the worst performance. Neural networks, interior points, and interior search methods all yield acceptable input power values in succession. The shared power of the proposed optimizer inverters is depicted in Fig. 12. As demonstrated, inverter 1 with a 0.7-Ω resistor shares the greatest power. In contrast, inverter 2, with a 1.4-Ω resistor, shares the least power for 3 kW and 5 kW in Fig. 12a and 7 kW and 10 kW in Fig. 12b.

**Figure 13:**
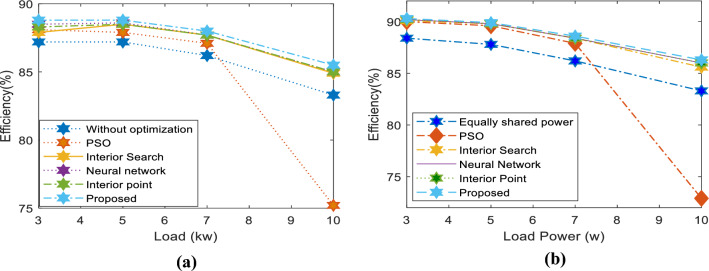
System efficiency in case of using different optimizers: (**a**) Case 1, (**b**) Case 2.

Figure 13a demonstrates the system's efficiency for loads of 3 kW, 5 kW, 7 kW, and 10 kW. The proposed system proves to be the most efficient among all the systems tested. For instance, at a 7-kW load, the proposed system achieves an efficiency of 88%, while the system without an optimizer has an efficiency of only 86%.

### Case 2

Three inverters, each with the parameters listed in Table 3, share loads of 3 kW, 5 kW, 7 kW, and 10 kW. In this case, two inverters have identical parameters. Like the first case, a comparison of optimizers is presented in Table 4. Across the four load scenarios, the proposed technique yields the lowest power losses of 322 W, 560 W, 897 W, and 1587 W, respectively. Without optimization, the losses are 395 W, 693 W, 1120 W, and 2002 W, respectively. The neural network, interior point, and interior search optimizers achieved the second-best results after the proposed optimizer. In contrast, the PSO optimizer produces high ripples and fails to capture the optimal solution for a 10-kW load.

**Table 3:** Case 2 inverters’ parameters.

System resistance and reactance	Inverter 1	Inverter 2	Inverter 3	DC input filter
Series resistance	0.7 W	2.1 W	0.7 W	0.5
Inductance	1 mH	3 mH	1 mH	0.4 mH
Voltage drop	1.6 V	3.2 V	1.6 V	–-
Capacitance	–-	–-	–-	800 µF

**Table 4:** Case 2 system power losses in the case of different optimizers.

Load power		Without optimization	PSO	Interior search	Neural network	Interior point	Proposed
3 kW	P_L_(w)	395	335	328	326	327	322
500 VAR	η (%)	88.4%	90%	90.1%	90.2%	90.2%	90.3%
5 kW	P_L_(w)	693	579	567	568	567	560
500 VAR	η (%)	87.8%	89.6%	89.8%	89.8%	89.8%	89.9%
7 kW	P_L_(w)	1120	962	913	914	913	897
500 VAR	η (%)	86.2%	87.9%	88.4%	88.4%	88.4%	88.6%
10 kW	P_L_(w)	2002	3715	1682	1624	1622	1589
500 VAR	η (%)	83.3%	72.9%	85.6%	86%	86%	86.3%
Optimizer execution time			464.9 ms	342.6 ms	693.4 ms	7.7 ms	

**Figure 14:**
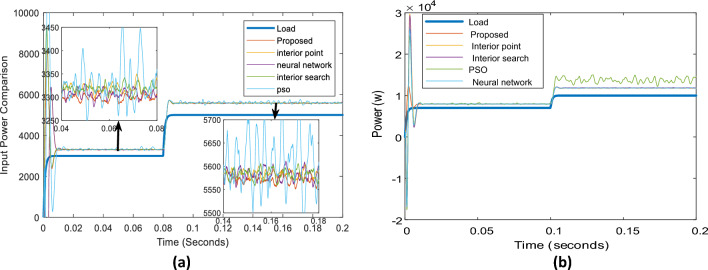
Parallel inverters Input power comparison in case of different optimizers: (**a**) in case of 3 kW and 5 kW loads, (**b**) in case of 7 kW and 10 kW load.

Figure 9b displays the phase A currents of the three inverters. As all optimizers demonstrate, the first and third inverters share the same current, while the proposed optimizer has the lowest 2nd inverter current value. The PSO optimizer continues to produce fluctuating current values. Figure 14 compares the input powers for the various optimizers. Figure 14a shows the optimizers' powers for a 3-kW load between 0 to 0.08 s and a 5-kW load between 0.08 to 0.2 s, while Fig. 14b illustrates the optimizers' powers for a 7-kW load between 0 to 0.1 s and a 10 kW load between 0.1 to 0.2 s. The figure reveals that the proposed optimizer produces the lowest power losses, while the PSO optimizer produces high fluctuating power.

**Figure 15:**
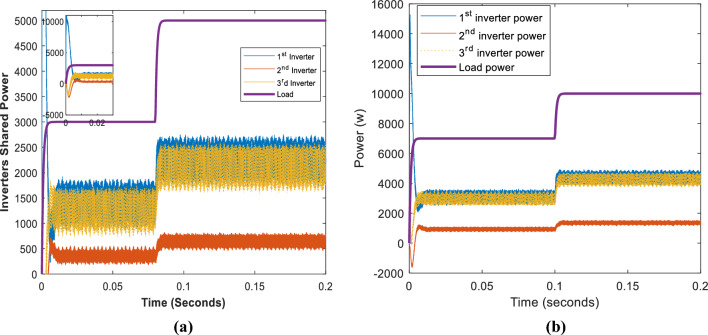
The three inverters shared powers: (**a**) in case of 3 kW and 5 kW load, (**b**) in the case of 7 kW and 10 kW.

Figure 15a illustrates the shared power between the three inverters for loads of 3 kW and 5 kW, while Fig. 15b shows the shared power for loads of 7 kW and 10 kW. The figure indicates that the first and third inverter share a more significant power than the second inverter.

As shown in Fig. 13b, all optimizers achieve high efficiency compared to equally shared currents under all load conditions. However, the proposed optimizer attains the highest efficiency among all tested methods. At a 10-kW load, the proposed optimizer achieves an efficiency of 86.3%, whereas the system efficiency without optimization is only 83.3%. The PSO optimizer results in minimal power loss, with an efficiency of only 72.9%.

### Case 3

In this case, inverters with identical specifications to those in Case 2 are utilized, except for the third inverter, which has an inductance of 2 mH instead of 1 mH. However, this change in inductance does not affect the optimal power losses. Table 5 shows that at a 7-kW load, the proposed optimizer yields minimal losses of 896 W and an efficiency of 88.6%. The proposed optimizer achieves the same results as in case 2, while all other optimizers exhibit a slight reduction in efficiency. The decrease in power losses observed in other optimizers is due to the decrease in current ripples resulting from the increase in the inductance filter. From this case, it can be deduced that the variation in inductance does not affect the minimum power losses.

**Table 5:** Case 3 system power losses in the case of different optimizers.

Load power		Without optimization	PSO	Interior search	Neural network	Interior point	Proposed
7 kW	P_L_(w)	1119	985	922	923.6	921	896
500 VAR	η (%)	86.2%	87.6%	88.4%	88.3%	88.4%	88.6%
10 kW	P_L_(w)	2001	3715	1683	1641	1638	1588
500 VAR	η (%)	83.3%	72.9%	85.6%	85.9%	85.9%	86.3%
Optimizer execution time			464.9 ms	342.6 ms	693.4 ms	7.7 ms	

Based on the analysis of the preceding three case studies, the displayed competence of the proposed optimizer is notably outstanding in its capacity to attain peak system efficiency when juxtaposed with all other optimizer variants. This enhancement in system efficiency denotes a substantial 3% increase compared to its performance under conditions of equally shared current. Compared to alternative optimizers, the proposed optimizer consistently maintains minimal fluctuations in inverter currents and their shared power. The system controller ensures the stability and reliability of the system under all load change scenarios.

Compared to other optimizers, the proposed approach is completed quickly. The interior point optimizer requires only 7.7 ms to complete. In comparison, the interior search, PSO, and neural network optimizers require 342.6, 464.9, and 693.4 ms, respectively. One significant advantage of the proposed optimizer is its shorter execution time and lower number of calculations compared to the other optimizers. The RLS estimator expedites system operation, attaining the actual parameter values in significantly less than 100 microseconds.

The control system presented with the optimizer provides a dependable solution for parallel-operating inverters, demonstrating various advantages such as rapid response times, system stability under diverse load conditions, high efficiency, efficient limiting circulating currents between inverters, and a minimal computational and memory footprint for the controller.

## Conclusion

This paper introduces an analytical optimizer designed to maximize the efficiency of parallel inverter systems while minimizing circulating current. A master–slave control configuration regulates the parallel inverter system. Optimized PID controllers regulate the system inverters, with the master inverter employing a PID voltage loop and the slave inverters utilizing a PID current loop. SPWM is applied to generate control signals for the inverter switches, and PSO optimization is employed to tune the PID parameters. The optimizer mathematical model is developed by analyzing power losses in the d-q model of the parallel inverter system. The optimizer uses an efficient RLS estimator to estimate system parameters. The results of the proposed optimizer are compared to those of PSO, neural network, interior search, and interior point optimizers, leading to the following conclusions:

- The proposed optimizer consistently delivers high efficiency, improving system efficiency by 3% compared to equally shared current scenarios.
- The proposed optimizer demonstrates a significantly reduced execution time compared to other optimizers, such as the neural network optimizer, which takes 693 ms.
- The utilization of a recursive least-square online estimator also accelerates system operations.
- Tuning PID parameters with PSO enables a simple and fast controller to attain high-performance levels.

The presented optimization technique has been thoroughly discussed, validated, and tested through simulation. While the validation was conducted using a three-parallel inverter system, the concept can readily be scaled to accommodate any number of phases.

In future work, integrating photovoltaic (PV) systems as power sources for inverters will be explored, aiming to enhance system efficiency and extract maximum power from the PV arrays. Additionally, a practical model of a three-parallel inverter system will be developed and empirically tested, providing a cost-effective control solution for industrial applications. Lastly, the investigation will extend to evaluating machine learning methodologies, such as Support Vector Machines or regression, as potential alternatives to the traditional PID controller, aiming to enhance the system's response.

## Data Availability

The datasets used and/or analysed during the current study available from the corresponding author on reasonable request.

## Abbreviations

PSO: Particle swarm optimization
PID: Proportional integrator derivative
SPWM: Sinewave pulse width modulation
PV: Photovoltaic
RLS: Recursive least squares

## List of symbols

V: Voltage
i: Current
${i}_{d}\&{i}_{q}\&{i}_{o}$: Direct and quadrature and zero currents
$${i}_{L}$$: Load current
${i}_{a}\&{i}_{b}\&{i}_{c}$: Three phase currents
N: Number of parallel inverters
K: Counter 1… N
$${K}_{p}$$: Proportional constant
$${K}_{i}$$: Integrator constant
$${K}_{d}$$: Differentiator constant
${e}_{t}$: Real-time error
R: Load equivalent resistance
L: Load equivalent inductance
C: Load parallel capacitor filter
${L}_{1}$: Inverter series inductor filter
${R}_{1}$: Inverter series loss resistance
${T}_{f}$: Is the filter time constant
${P}_{i}$: Position of I particle
${v}_{i}$: Speed of I particle
$$w$$: Inertia weight
${P}_{local\,best\left(i\right)}$: The best position of I particle
${P}_{global\,best\left(i\right)}$: The best position in the swarm
$${r}_{1}{\& r}_{2}$$: Random numbers
$${c}_{1}\& {c}_{2}$$: Acceleration coefficients
$\Upsilon$: Value of around 0.01
${V}_{t}$: Switch voltage drop
${F}_{opt}$: System loss function needs to be minimized
$${\psi }_{t}$$: Coefficient matrix
$$\beta$$: Parameters matrix
${Y}_{measured}$: Measured data matrix
PL(w): Power losses (watt)
η (%): Efficiency
